# An overview of the development of perinatal stress-induced fatty liver and therapeutic options in dairy cows

**DOI:** 10.1007/s44154-024-00206-5

**Published:** 2025-02-24

**Authors:** Haitao Hu, Lamei Wang, Rui Zhang, Mei Tian, Shuo Zhang, Hongrui Li, Chuanjiang Cai, Junhu Yao, Jianguo Wang, Yangchun Cao

**Affiliations:** 1 College of Animal Science and Technology, Northwest A&F University, Xianyang, 712100 China; 2 College of Veterinary Medicine, Northwest A&F University, Xianyang, 712100 China

**Keywords:** Periparturient cows, Fatty liver, Glucolipid metabolism, Nutritional additives

## Abstract

This review summarizes the mechanisms of hepatic glycolipid metabolism disorders caused by the negative energy balance encountered in periparturient dairy cows and the relevant research on nutritional additives as a therapeutic option. Factors such as dietary management, hormonal regulation, and overall metabolic stress in the body of the transition cow all contribute greatly to fatty liver formation. Nutritional strategies, such as using gluconeogenic precursors, growth factor, natural plant extracts, and methyl donors can positively modulate the negative effects of fatty liver in periparturient dairy cows. Choline, a methyl donor as a feed additive in transition cows minimizes lipid accumulation in the liver by increasing the efficiency of lipoprotein transport. In conclusion, the disruption of hepatic gluconeogenesis, changes in hormone levels, oxidative stress, and endoplasmic reticulum stress during the transition period in dairy cows collectively disturb hepatic anabolic homeostasis. This disruption promotes the formation of fatty liver and reduces lactation performance in dairy cows. Understanding the specific physiological phenomena of hepatic lipid metabolism disorders in transition cows and intervening with nutritional additives will reduce the negative effects of transition stress and improve animal health.

## Introduction

The period of 21 days before and 21 days after calving is known as the periparturient period of the dairy cow, also called the transition period. During the periparturient period, the cow's body metabolism undergoes the most drastic strikes and changes during their growth stage. The main reason was a dramatic increase in energy requirements due to the rapid growth of the fetus at the stage of coming up to calving, the renewal of mammary tissue, and the initiation of lactation (Buttchereit et al. 2012). On the other hand, the volume of the abdominal cavity occupied by the fetus and the changes in circulating hormone levels in cows lead to a decrease in the appetite of transition cows (Grummer et al. 2004). As a result, the energy gained through forage was not sufficient to satisfy the maintenance and lactation needs of the periparturient cow, which in turn triggered the mobilization of adipose tissue to provide energy, resulting in the phenomenon of negative energy balance (Wathes et al. 2007; LeBlanc. 2012). Notably, large quantities of non-esterified fatty acids (NEFA) were produced following this process, which were ultimately metabolized in the liver along with the body's blood circulation (Huang et al. 2024; Duffield et al. 2009).

Ketosis in periparturient dairy cows was a metabolic disease that was closely related to liver lipid deposition (Chapinal et al. 2012). When the amount of NEFA circulated through the blood into the liver exceeded the liver's capacity for oxidative catabolism and the secretion rate of very low-density lipoprotein (VLDL) transported triglycerides, hepatic lipid deposition was triggered (White. 2015). Both mitochondria, the center of energy metabolism, and the endoplasmic reticulum, a key site of lipid synthesis, also incurred oxidative stress to maintain normal cellular function when the liver cells were challenged with glycolipid metabolism (McFadden. 2020). However, the root cause of all the adverse effects of the negative energy balance in transition dairy cows that disturbed the cellular metabolism of the liver was caused by the breakdown of the homeostasis of glucose and lipid metabolism in the liver.

The extent of fat mobilization in dairy cows was found to be linked to dry matter intake, negative energy balance, and plasma concentrations of glucose and ketone bodies (Weber et al. 2013b; Hammon et al. 2009). High concentrations of NEFA during the transition period leads to disruptions in hepatic metabolism and potentially metabolic disorders that negatively impact lactation performance. Nutritional strategies aim to elevate blood levels of propionic acid and provide gluconeogenic precursor substances to compensate for glucose deficiency in energy-deficient cows. Nutritional strategies for transition cows involve providing gluconeogenic precursors and growth factors that regulate glycolipid metabolism (Zhang et al. 2020; Bobe et al. 2007). Additionally, natural plant extracts added to dairy cow feed may offer benefits beyond nutritional value, including antioxidant properties and enhanced immune metabolism (Lopreiato et al. 2020). Another approach involves supplementing dairy cow feed with methyl donors to promote lipid metabolism (Abbasi et al. 2017). The addition of choline, a methyl donor feed additive for transition cows, reduces lipid accumulation in the liver by enhancing lipoprotein transport efficiency (Lopreiato et al. 2020; McFadden et al. 2020). Although progress has been made in understanding the pathological features and negative impact of ketosis on dairy cows' performance, a comprehensive description of the multifaceted causes of hepatic lipid deposition in periparturient dairy cows and effective treatments is lacking. Therefore, this paper aims to provide an overview of fatty liver occurrence in transition cows by considering homeostatic mechanisms of hepatic glucose-lipid metabolism, changes in defense responses, and intracellular regulatory networks associated with negative energy balance. Additionally, possible therapeutic options for transition cows adapting to physiological challenges will be prospectively evaluated and summarized.

## General changes in metabolism during the transition period

Research has shown that elevated levels of NEFA and concentration of β-hydroxybutyric acid in serum of dairy cows during the pre-partum and post-partum periods posed a pregnancy risk as well as affecting milk production (Ospina et al. 2010a; McArt et al. 2013). In response to dysregulated energy metabolism, dairy cow organisms underwent a combination of adaptive mechanisms including the endocrine and immune systems. The combined effect of all these challenges was a deterioration in fertility and milk production, which led to reduced profitability (Esposito et al. 2014). Other perinatal diseases such as retained placenta, uterine infections, and altered estrous cycles accompany ketosis in dairy cows (Ospina et al. 2010b, Raboisson et al. 2014, Abdelli et al. 2017). An interesting finding was that the health status of cows was directly related to greenhouse gas emissions, the subclinical ketosis cows resulted in altered gastrointestinal fermentation and increased CO_2_ emissions (Mostert et al. 2018, Pechová and Nečasová. 2018).

Dairy cows susceptible to ketosis had reduced metabolism of vitamins and coenzymes, decreased synthesis of secondary metabolites of lipids, carbohydrates, and polysaccharides, and impaired gluconeogenic processes with lower concentrations of key hepatic glycoconjugate enzymes in the liver (Shahzad et al. 2019). Proteins associated with fatty acid oxidation, glycolysis, electron transfer, protein degradation, antigen processing, and cytoskeletal rearrangement were down-regulated in the liver when dairy cows were faced with negative energy balance. Meanwhile, the expression of glycolytic enzymes was positively correlated with glucose/glycogen content, and the expression of β-oxidase was negatively correlated with total liver fat content (Kuhla et al. 2009).

### Crosstalk between liver glucose and lipid metabolism in transition cows

Glucose in the bloodstream serves as a vital substrate for energy metabolism and synthetic pathways in all animal cells and organisms, including fatty acid oxidation and lipid synthesis (Gogga et al. 2011). Specifically, for ruminants transitioning to consuming fibrous carbohydrate feed post-weaning, liver gluconeogenesis becomes the primary source of blood glucose (Zhang et al. 2015). After feed intake by ruminants, volatile fatty acids (VFA) are produced by fermentation of feed in the rumen under the action of microorganisms and various digestive enzymes, which are then absorbed into the bloodstream. Among these VFAs, propionic acid is a major substrate for liver gluconeogenesis in ruminants (Larsen and Kristensen. 2009). It is worth noting that feed intake in periparturient ruminants is negatively correlated with the production of NEFA (Kuhla et al. 2016). Contrarily, the significant accumulation of ketone bodies in the bloodstream resulting from the incomplete oxidation of surplus NEFA diminishes the appetite of dairy cows. This reduction in appetite may lead to a decrease in the precursors utilized for gluconeogenesis in the liver (Allen et al. 2009). This creates a detrimental cycle contributing to the dysregulation of blood glucose levels in periparturient cows, often manifesting as decreased blood glucose levels (Kupczynski et al. 2011; Mecitoglu et al. 2016). Fatty acid oxidation yields acetyl coenzyme A (acetyl-CoA), which combines with oxaloacetate to produce citrate, entering the tricarboxylic acid (TCA) cycle. However, during times of heightened glucose demand, oxaloacetate is increasingly diverted towards gluconeogenesis, preventing the complete oxidation of acetyl-CoA, leading instead to the production of ketone bodies, primarily acetone, acetoacetate, and β-hydroxybutyric acid. Excess fatty acids may also undergo re-esterification in the liver, being stored as triglycerides. Nonetheless, the export of surplus triglycerides from liver cells is restricted due to ruminants' limited production capacity for VLDL (Schäff et al. 2012, 2013).

During the transition period, there's a dynamic interplay between liver gluconeogenesis for glucose production and the mobilization of body fat for energy generation in dairy cows. Fat mobilization affects the expression of genes involved in gluconeogenesis, fatty acid oxidation, ketogenesis, and cholesterol synthesis in the liver, as well as the activity of transcription factors related to energy metabolism (Weber et al. 2013a). Furthermore, the impact of heightened body fat mobilization in early lactation on the expression of gluconeogenesis-related genes outweighs that of genes related to lipid metabolism, indicating the dependency of liver glucose metabolism on hepatic lipid status and fat mobilization during this period (Abuelo et al. 2020).

### The gluconeogenesis pathway is impaired in fatty liver cows

The mobilization of fat in transition dairy cows may significantly influence feed intake regulation (Ingvartsen. 2006). Reduced feed intake leads to decreased propionic acid production from rumen fermentation in dairy cows, resulting in insufficient precursors for gluconeogenesis in the liver (Weber et al. 2013b). Consequently, this deficiency is a key driver for mobilizing reserve lipolysis in transition cows, thereby promoting the formation of fatty liver. Severe ketosis of cows after calving resulted in decreased liver pyruvate carboxylase (PC) and Phosphoenolpyruvate carboxykinase (PEPCK) expression. Interestingly, fatty acid content did not exclusively influence the alteration in PC expression (Abuelo et al. 2020). Monitoring PC and PEPCK mRNA expression levels and enzyme activities could be beneficial in detecting subclinical ketosis in cows (Wang et al. 2012). Overfeeding induced fatty liver in cows before parturition, with results indicating that the overfed group had lower hepatic gluconeogenesis capacity compared to the prepartum restricted-feeding group. Cows with fatty liver exhibited reduced activity of key gluconeogenic enzymes in the liver, leading to inadequate glucose production after parturition (Zhang et al. 2023b).

The rate of liver gluconeogenesis in dairy cows depends on the activity of several key catalytic enzymes. For instance, PEPCK serves as the rate-limiting enzyme for pyruvate's preferential entry into the gluconeogenesis reaction. Additionally, Phosphofructokinase 2 (PFK2) and glucose 6-phosphatase are crucial enzymes for the glucose release step (Aschenbach et al. 2010). High circulating levels of NEFA inhibit the expression of PC and PEPCK mRNA in dairy hepatocytes, resulting in decreased gluconeogenesis efficiency and promoting a negative energy balance (Li et al. 2012). Genome-wide association analysis investigated polymorphisms of differential genes in cows with varying degrees of negative energy balance, revealing the PC gene's significant potential in determining events predisposing transition cows to ketosis. This finding not only holds implications for genetic breeding but also underscores the essential role of PC in the development of ketosis in dairy cows (Soares et al. 2021b).

### Hormonal molecules in the homeostasis of glycolipid metabolism

Hormones function as second messenger molecules that mediate the metabolic changes experienced by transition cows. There exists a complex crosstalk between adipose tissue and liver glucose metabolism in transition dairy cows, involving numerous hormones and adipokines. Transition cows opt to redistribute nutrients to support milk production and undergo a phase of physiological insulin resistance, accompanied by negative energy balance, lipolysis, and weight loss (Leblanc. 2010). Insulin resistance induces type II ketosis in dairy cows, characterized by elevated blood glucose levels similar with type 2 diabetes mellitus. This type of ketosis suggests a disruption in glucose utilization in the body and is linked to abnormal liver function and oxidative stress (Zhang et al. 2019). The expression of liver insulin receptors diminishes significantly in dairy cows with fatty liver, indicating reduced responsiveness to insulin (Liu et al. 2010). Research indicates that significant weight loss in cows before and after calving correlates with increased breakdown of adipose tissue due to insulin resistance (Zachut et al. 2016).

Adipocytes or adipose tissues regulate gene expression and secrete specific hormones to maintain the metabolic equilibrium required for positive or negative energy balance, typically involving adipokines like adiponectin and leptin (Stern et al. 2016). Changes in both blood glucose levels and lipid mobilization occur in dairy cows experiencing ketosis during the transition period, and these changes correlate with fluctuations in serum levels of adiponectin and leptin (Shen et al. 2020). Adiponectin inhibits the expression of genes involved in hepatic lipogenesis and cholesterol synthesis by binding to the adiponectin receptor, subsequently initiating the LKB/AMP/AMPK pathway, thereby reducing hepatic lipid accumulation (Awazawa et al. 2009). Under conditions of elevated blood NEFA, adiponectin enhances insulin sensitivity and may function as a hormone to regulate negative energy balance and blood NEFA levels in dairy cows (Mecitoglu et al. 2016).

### The association between fatty liver and stress in transition dairy cows

During the periparturient period, dairy cows often experience nutrient and energy deficiencies, necessitating adaptive regulation of liver metabolism to cope with metabolic stress (Pascottini et al. 2020). This adjustment is crucial as the heightened concentration of NEFA can trigger the production of reactive oxygen species (ROS) and endoplasmic reticulum stress, thereby increasing the susceptibility of periparturient dairy cows to developing fatty liver (Li et al. 2023). Furthermore, disturbances in lipid metabolism within the liver of dairy cows with mild to moderate fatty liver are closely associated with oxidative stress, and severe oxidative stress can potentially lead to hepatic fibrosis in transition cows (Zhang et al. 2023a; Sejersen et al. 2012).

## Fatty liver and oxidative stress

Oxidative stress in transition dairy cows has been strongly correlated with triglyceride deposition in the liver (Wu et al. 2020). During the early postpartum period, cows experience oxidative stress and downregulation of apolipoprotein B100 (ApoB 100) in the liver, which is consistent with liver lipid accumulation, suggesting a potential link between oxidative stress and ApoB100 degradation in dairy cows (Bernabucci et al. 2009). The consequences of liver fat deposition can induce oxidative stress by affecting multiple mechanisms of ROS generation. For example, NEFA-induced lipid accumulation impairs the oxidative capacity of mitochondria and stimulates the peroxisomal pathway of lipid oxidation in bovine primary hepatocytes, directly leading to ROS production and oxidative stress (Shi et al. 2015; Spahis et al. 2017). The excessive production of ROS causes a reprogramming of lipid metabolism and alters insulin sensitivity (Chen et al. 2020). Peroxisome proliferator-activated receptor-γ (PPAR-γ) may participate in or regulate hepatic fat deposition in dairy cows as a key genetic factor by influencing biological processes such as insulin resistance, gluconeogenesis, oxidative stress, and inflammation (Shi et al. 2020).

Research in both clinical and animal models of non-alcoholic fatty liver disease (NAFLD) has consistently shown a strong correlation between fat formation and the presence of oxidative stress. Multiple studies have indicated that heightened oxidative stress in transitioning dairy cows can result in ketosis, fatty liver, and immune dysfunction (Song et al. 2021; Shi et al. 2015, Sordillo and Aitken. 2009). Additionally, reducing oxidative status through nutritional management has been found to improve the severity of various metabolic and infectious diseases in transition dairy cows, further emphasizing the impact of oxidative stress on disease development (Batistel et al. 2018; Zhou et al. 2016).

## Fatty liver and endoplasmic reticulum stress

If oxidative stress continues to increase unchecked, it can result in the overproduction of ROS, leading to endoplasmic reticulum stress (Xiang et al. 2020). Additionally, during early lactation, dairy cows face various metabolic and inflammatory challenges, including NEFA, TNF-α, IL1-β, ROS, and LPS, which are implicated in triggering the endoplasmic reticulum stress response (Ringseis et al. 2015). The endoplasmic reticulum, as the central organelle in the cell, regulates the primary synthesis of proteins and lipid biosynthesis. Studies on periparturient dairy cows have demonstrated that marker genes for endoplasmic reticulum stress are induced by free fatty acids from lipolysis early in calving, leading to concurrent endoplasmic reticulum stress and fatty liver development (Khan et al. 2015). Furthermore, experiments with primary hepatocytes from dairy cows in vitro have shown that NEFA treatment activates the endoplasmic reticulum calcium pathway, inducing endoplasmic reticulum stress and resulting in lipid accumulation in isolated hepatocytes (Li et al. 2021).

**Fig. 1:**
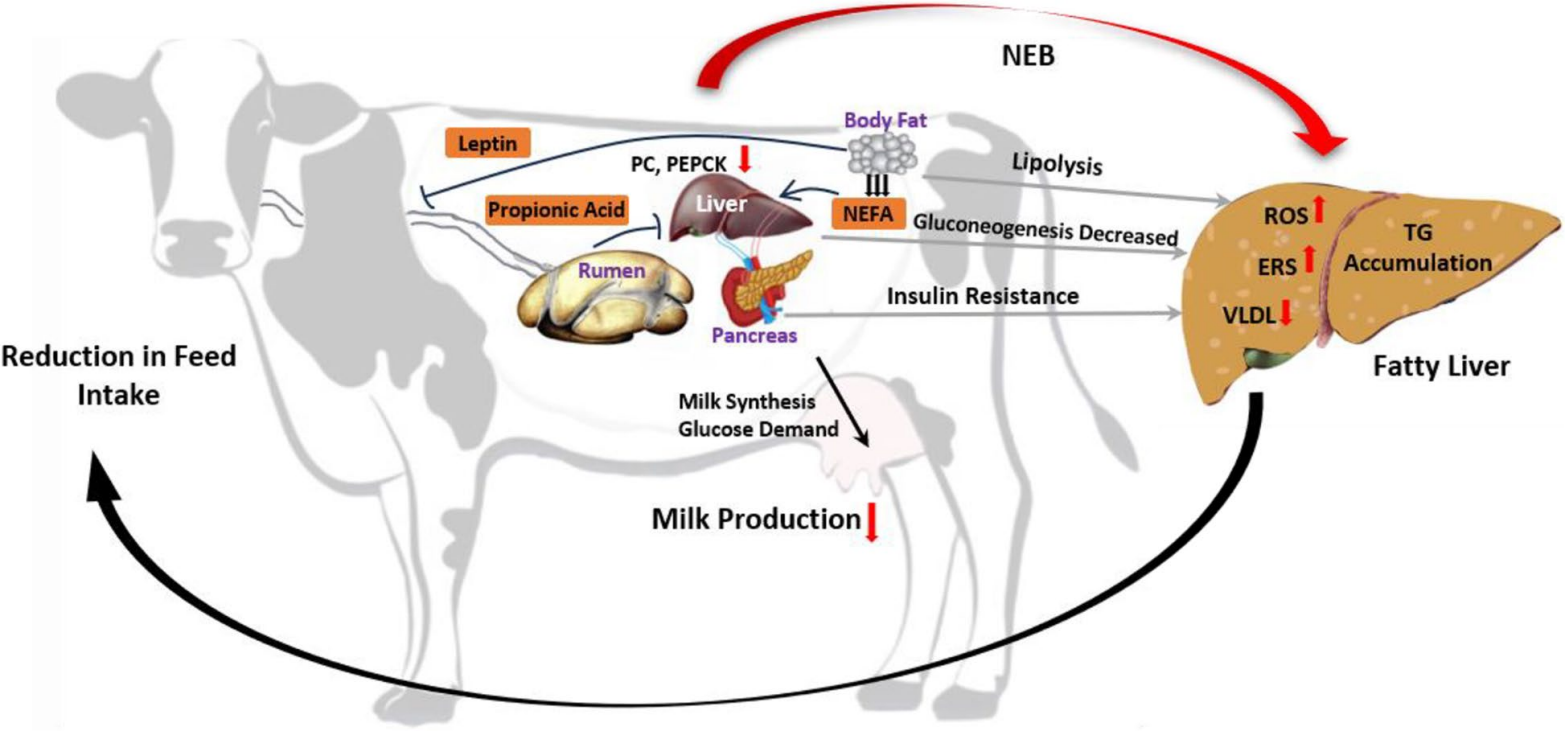
Summary of factors influencing fatty liver development in periparturient dairy cows. PC: Pyruvate carboxylase; PEPCK: Phosphoenolpyruvate carboxykinase; NEB: negative energy balance; NEFA: non-esterified fatty acids; ROS: reactive oxygen species; ERS: endoplasmic reticulum stress; TG: triglyceride; VLDL: very low-density lipoprotein.

High phosphorylation levels of unfolded protein response proteins such as protein kinase RNA-like ER kinase (PERK), activating transcription factor-6 (ATF6), inositol requiring protein-1α (IRE-1α), and glucose-regulated protein 78 (GRP78) indicated severe endoplasmic reticulum stress in fatty liver cows, suggesting its involvement in the progression of fatty liver in dairy cows (Zhu et al. 2019). Furthermore, IRE-1α and c-Jun N-terminal kinase (JNK) were found to be activated in the livers of transition cows. In vitro experiments demonstrated that targeting the IRE1α-JNK axis could reduce NEFA-induced lipid accumulation in bovine hepatocytes by regulating lipogenesis and fatty acid oxidation (Gao et al. 2023). Additionally, activation of the PERK signaling pathway during the periparturient period in dairy cows significantly enhanced the liver's adaptive response to high plasma concentrations of NEFA, effectively regulating hepatic lipid metabolism and reducing lipid accumulation (Huang et al. 2021). It was noted that β-hydroxybutyric acid-induced insulin resistance was ameliorated when endoplasmic reticulum stress was suppressed, suggesting a potential strategy to improve hepatic resistance to insulin resistance and other liver diseases in ketotic cows through endoplasmic reticulum stress (Lei et al. 2021). The causes of fatty liver formation in periparturient dairy cows are complex, and several possible factors mentioned in this review are summarized in Fig. 1.

### The role of genetic factors in susceptibility to fatty liver in dairy cows

In addition to the factors mentioned above, genetic markers and breed differences are also closely related to the susceptibility of dairy cows to fatty liver. Differential genes of transitional dairy cow liver determined the development of fatty liver, glutathione metabolism play a key role in regulating susceptibility, while arachidonic acid metabolism is involved in resistance to disorders of hepatic lipid metabolism in dairy cows (Pralle et al. 2021). Different SNPs in the cow genome significantly impact ketosis. Potential candidate genes related to lipid metabolism, insulin regulation, and immune response also play a role in the progression of ketosis. These genes influence the status of negative energy balance in transition cows (Klein et al. 2019; Gaddis et al. 2018). The researchers identified potential candidate genes associated with clinical and subclinical ketosis in the cow genome, such as acetyl-CoA acetyltransferase 2 (ACAT2) and insulin-like growth factor-1 (IGF1). Enrichment analyses of these genes highlighted key molecular functions and biological processes, including fatty acid metabolism, lipid metabolism, and inflammatory responses in dairy cows (Soares et al. 2021a). During the transition period, Simmental cows recovered their body condition more quickly, while Holstein–Friesian cows had higher milk production but were less able to restore their negative energy balance. In contrast, Holstein–Friesian cows showed lower liver function efficiency due to higher levels of leptin in their blood, which has an anorectic effect (Straczek et al. 2021).

### Feed additives to alleviate disorders of liver glycolipid metabolism in transition cows

#### Gluconeogenic precursors and growth factors

Improving glucose supply through exogenous additives can address glucose deficiency in transition cows. Supplementing transition cows with rumen glucose significantly influences carbohydrate metabolism and amino acid metabolic pathways, thereby alleviating lipolysis. This regulation may be achieved by impacting rumen flora, fecal microbial composition, and microbial metabolism (Wang et al. 2022, 2021). Propionic acid serves as the primary gluconeogenic precursor in dairy cows, and calcium propionate, which can be hydrolyzed in the rumen to propionic acid and Ca^2+^, holds promise as a beneficial feed additive for alleviating negative periparturient energy balance in transition cows (Zhang et al. 2020). Transition cows fed with calcium propionate showed higher lactose synthesis in the mammary glands compared to other groups, and milk production parameters such as milk yield, milk protein, and milk fat were also increased (Martins et al. 2019).

Administering glucagon subcutaneously to dairy cows at 8 days postpartum did not negatively affect lipid transport in plasma. This approach could be a viable option for effectively preventing or treating fatty liver in early lactation dairy cows (Duffield et al. 2003). External glucagon mitigated some of the adverse effects of mild fatty liver on the health and reproductive performance of transition dairy cows, thereby enhancing the overall performance and profitability of lactating dairy cows (Bobe et al. 2007). Glucagon significantly promoted glucagon receptor expression, activating the AMPK signaling pathway, increasing lipid oxidation and VLDL assembly in bovine hepatocytes, thereby decreasing hepatic fat accumulation (Li et al. 2019). Impairment of IGF1 synthesis is involved in the development of fatty liver in dairy cows. Various concentrations of IGF1 increased hepatocyte ApoB100, apolipoprotein E (ApoE), microsomal triglyceride transfer protein (MTTP), and low-density lipoprotein receptor (LDLR) mRNA abundance, ultimately leading to increased VLDL assembly (Li et al. 2016).

#### Potential natural plant extracts and prebiotics

Using functional nutritional additives, such as natural plant extracts and prebiotics, in transition cows may improve negative energy balance and reduce the harmful effects of stress through their active mechanisms (Table 1). Lonicera japonica extract can prevent metabolic diseases in transition cows, mainly by increasing dry matter intake, milk production, and stress tolerance (Zhao et al. 2020). Polyphenols, found in many botanical products such as green tea and curcumin, have beneficial effects on livestock health. These products alleviate metabolic stress in the liver, improve milk production performance, and prevent fatty liver syndrome in early lactation cows (Winkler et al. 2015). Quercetin, a flavonoid present in all plant species, reduced plasma aminotransferase and glutamate dehydrogenase in transition cows with quercetin supplementation, indicating a decrease in liver injury (Stoldt et al. 2015). Oregano extract (OE) significantly increased dry matter intake in early lactation Jersey cows but had no effect on disease incidence. OE and green tea extract (GT) did not significantly affect blood immune traits in transition cows, but they did reduce some oxidative stress biomarkers without affecting production traits (Vizzotto et al. 2021; Bosco Stivanin et al. 2019). During the transition period, feeding rumen-protected capsicum oleoresin resulted in higher fat-corrected milk yield, and improved milk fat and feed efficiency, without significantly impacting serum metabolites in dairy cows (Takiya et al. 2023). Research suggests that essential oils like curcumin oleoresin, garlic extract, or capsicum oleoresin had minimal or no effect on hemocytes, nutrient digestibility, antioxidant status, and hepatic mRNA expression in transition cows. However, they stimulated CD4 cell activation and subsequent immune effects (Oh et al. 2013). Adding plant extracts with antioxidant and anti-inflammatory properties during the transition period has shown potential for improving milk production performance and health parameters in postpartum cows. Further research is needed to fully understand the effects and mechanisms of action of plant bioactive compounds on transition cows.

**Table 1:** Effect of plant extracts and prebiotic products on transition cows

Nutraceutical	Treatments	Main outcomes	Reference
Lonicera Japonica Extract	1 g/kg and 2 g/kg Dry matter	Increased dry matter intake; improved lactation performance; enhanced anti-inflammatory and antioxidant capacity	(Zhao et al. 2020)
Plant Product Consisting of Green tea(95%) and Curcuma Extract(5%)	0.175 g of the Plant Product Per Kg of Dry Matter	Increased milk production in dairy cows; prevented fatty liver syndrome	(Winkler et al. 2015)
Quercetin	100 mg of Quercetin Dihydrate per kilogram of body weight	Reduced liver damage	(Stoldt et al. 2015)
Oregano Extract (OR) and Green Tea Extract (GT)	10 g per day of OR and 5 g per day of GT	Increased feed intake; OR and GT reduced oxidative stress biomarkers	(Vizzotto et al. 2021; Bosco Stivanin et al. 2019)
Grape Marc Meal Extract (GSGME)	Total Mixed Ration Supplemented with 1% of GSGME	Increased milk production	(Gessner et al. 2015, 2017)
Capsicum Oleoresin	100 mg/d	Increased fat-corrected milk production; improved milk fat and feed efficiency	(Takiya et al. 2023)
Essential Oil	2 g/d	CD4 cells were activated	(Oh et al. 2013)
Brazilian Green Propolis	25 g/d	Decreased inflammation and reduced lipid mobilization	(Kumprechtova et al. 2022)
Yeast and Glycerol-enriched Yeast Culture (GY)	2 L/d of GY (75.8 g/L glycerol and 15.3 g/L yeast)	Increased milk protein and milk fat levels; reduced plasma ketone body levels; promoted gluconeogenesis	(Ye et al. 2016)
yeast preparation	10 g/day5	Improved dry matter intake and milk production	(Lopuszanska-Rusek and Bilik. 2011)
Live Yeast	2.5 g/d/cow pre-calving and 10 g/d/head post-calving	Increased liver glycogen	(Al Ibrahim et al. 2010)
Live Yeast	1 × 10^10^/d/cow	Facilitated dietary transition and promoted the expression of anti-inflammatory and barrier genes in the rumen epithelium	(Bach et al. 2018)

Supplementing dairy cows in early lactation with dietary yeast culture supplements increases milk fat and protein levels. Glycerol-enriched yeast culture or glycerol supplements improve metabolic parameters by reducing plasma glucose, β-hydroxybutyric acid, and NEFA concentrations, while up-regulating hepatic gluconeogenesis enzymes in transitioning cows (Ye et al. 2016). Yeast preparations increased dry matter intake and milk yield in transition cows. Additionally, they tended to improve the cows' blood metabolic profile, notably lowering β-hydroxybutyric acid (BHBA) levels and reducing aspartate aminotransferase (AST) activity (Lopuszanska-Rusek and Bilik. 2011). Live yeast enhances the adaptation of rumen epithelium during dietary transition and inhibits the inflammatory response by reducing tumor necrosis factor α and increasing defensin β1 expression (Bach et al. 2018).

#### Choline

Methyl donors are essential for single-carbon metabolism in dairy cows, contributing to the synthesis of lipids, nucleotides, proteins, and the maintenance of redox homeostasis. Choline in the liver contributes to the production of betaine, phosphatidylcholine, and acetylcholine (Pizzo et al. 2017). These choline-derived substances serve various biological functions, such as constituting biofilm components, facilitating information transfer, participating in methyl metabolism, and promoting fat metabolism and brain development (Irvine et al. 2022; Zeisel. 2006). By feeding transitioning cows rumen-protected choline, betaine synthesis is enhanced as a methyl donor, leading to the re-methylation of homocysteine (Esposito et al. 2014). The results demonstrate an upward trend in milk yield and fat-corrected milk, indicating improved liver fatty acid metabolism and enhanced early lactation performance in dairy cows (Pinotti et al. 2003; Holdorf et al. 2023).

**Fig. 2:**
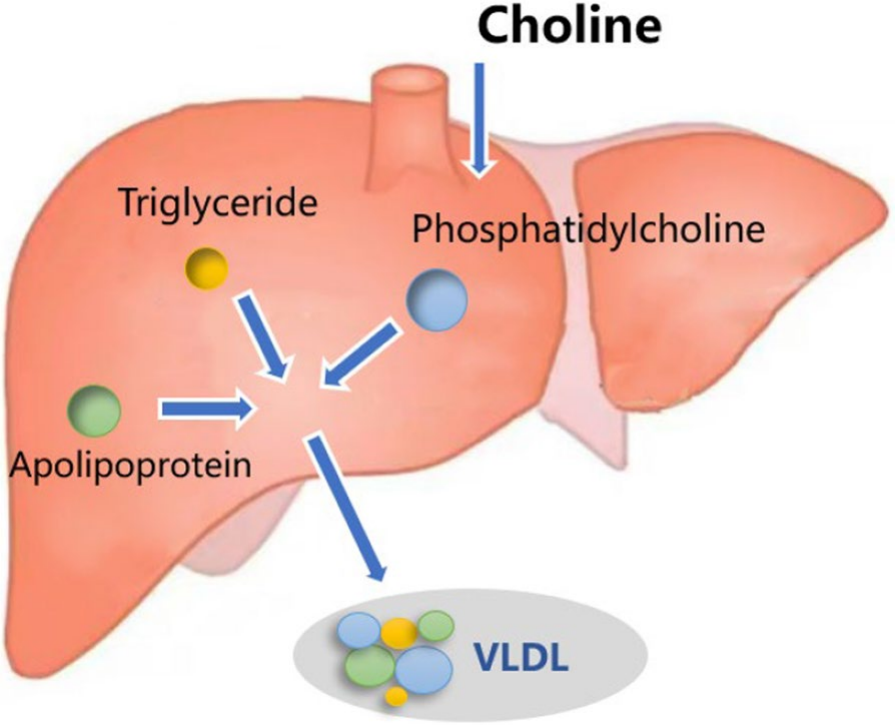
The pathway of choline action in the treatment of fatty liver in dairy cows. VLDL: very low-density lipoprotein

Choline is a crucial nutrient for dairy cows during the transition period. It plays a vital role in alleviating fatty liver, reducing serum NEFA levels, increasing phosphatidylcholine synthesis, maintaining lactation or physiological functions, and improving antioxidant activity (Hansen et al. 2016; Goselink et al. 2013; Abbasi et al. 2017). In vitro studies using primary hepatocyte cell lines from cows have shown that adding different concentrations of choline increases the complete oxidation of pyruvate, decreases the production of β-hydroxybutyrate, reduces ROS production, and tends to decrease triglyceride production in hepatocytes (Chandler et al. 2020). Reduced phosphatidylcholine content can exacerbate lipid accumulation in the liver by limiting lipoprotein transport (Cole et al. 2012; Vance. 2008). Impairing hepatic phosphatidylcholine biosynthesis significantly decreases circulating levels of VLDL and HDL (Robichaud et al. 2009). Lower levels of phosphatidylcholine in newly synthesized VLDL particles result in ineffective functioning and easy absorption after entering circulation (Cole et al. 2012). These findings suggest that feed additives that regulate phosphatidylcholine synthesis in the liver can promote the synthesis and production of VLDL in the hepatic secretory pathway. This, in turn, accelerates triglyceride transport and aids in the treatment of fatty liver disease in periparturient cows. Figure 2 illustrates the pattern of choline treatment for fatty liver in transition cows. This treatment promotes phosphatidylcholine synthesis in the liver, facilitates the assembly of VLDL, and accelerates triglyceride transport.

#### Methionine and betaine

Supplementing transition cows with rumen-protected methionine (RPM) has been shown to have positive effects on their overall health and milk production. Studies have indicated that RPM supplementation increases dry matter intake, milk yield, and milk protein synthesis rate in dairy cows, helping transition cows overcome physiological challenges in a healthier manner (Elsaadawy et al. 2022, Han et al. 2018). Transition cows supplemented with RPM showed increases in milk fat, total solids content, and the fat-to-protein ratio by 0.48%, 0.66%, and 0.09%, respectively. Additionally, total milk protein content increased by 0.13% (Michelotti et al. 2021). The inclusion of RPM in the diet of postpartum cows not only increases dry matter intake but also regulates hepatic lipid metabolism, improves postpartum lactation performance, enhances antioxidant capacity and immune function in transition cows, thereby promoting energy balance (Hansen et al. 2016). Betaine, a natural oxidation product of choline found in plants, has been found to enhance milk production in mid-lactation dairy cows by promoting feed digestion, especially when methionine supply is limited (Wang et al. 2010). The addition of betaine also increases antioxidant capacity and serum glucose concentration in dairy cows (Dunshea et al. 2019; Shah et al. 2020). However, feeding transition cows with liquid supplements containing betaine has been shown to improve lactation performance but increase adipose tissue mobilization and ketone body production in early lactation (Monteiro et al. 2017). Overall, adding methyl donors can help alleviate stress damage in transition cows, although we still have a limited understanding of how liver cells in transition cows utilize methyl donors.

## Conclusions and future work

Fatty liver is a major concern for dairy cow welfare and is caused by metabolic stress during the transition period. The disruption of hepatic glucose-lipid metabolism in transition cows leads to the formation of fatty liver, which is influenced by hormonal molecules, oxidative stress, endoplasmic reticulum stress, and genetics. Researchers have found that natural plant extracts, prebiotics, and methyl donors can improve the health and performance of transition cows. Future studies should focus on evaluating the combined effects of multiple nutritional additives that have shown therapeutic potential for fatty liver, exploring their effectiveness as comprehensive treatments. Additionally, from a genetic selection perspective, selecting animals less prone to fatty liver is crucial. The article reviews the mechanisms of fatty liver formation and summarizes effective nutritional additives. It aims to provide new perspectives for further research on alleviating fatty liver, which is induced by negative energy balance stress during the transition period in dairy cows.

## Data Availability

Not applicable.

## Abbreviations

NEFA: Non-esterified fatty acids
VLDL: Very low-density lipoprotein
VFA: Volatile fatty acids
TCA: Tricarboxylic acid
Acetyl-CoA: Acetyl coenzyme A
PC: Pyruvate carboxylase
PEPCK: Phosphoenolpyruvate carboxykinase
PFK2: Phosphofructokinase 2
ROS: Reactive oxygen species
ApoB 100: Apolipoprotein B100
PPAR-γ: Peroxisome proliferator-activated receptor-γ
NAFLD: Non-alcoholic fatty liver disease
PERK: Protein kinase RNA-like ER kinase
ATF6: Activating transcription factor-6
IRE-1α: Inositol requiring protein-1α
GRP78: Glucose-regulated protein 78
JNK: C-Jun N-terminal kinase
IGF1: Insulin-like growth factor1
ACAT2: Acetyl-CoA Acetyltransferase 2
ApoE: Apolipoprotein E
MTTP: Microsomal triglyceride transfer protein
LDLR: Low-density lipoprotein receptor
OE: Oregano extract
GT: Green tea extract
BHBA: β-Hydroxybutyric acid
AST: Aspartate aminotransferase
HDL: High-density lipoprotein
RPM: Rumen-protected methionine
